# University of Louisville—Preliminary Course

**Published:** 1853-10

**Authors:** 


### UNIVERSITY OF LOUISVILLE—PRELIMINARY COURSE.

The informal course of lectures in the University of Louisville will commence on Monday, the 3d inst., at which time the anatomical rooms will also be open. The course will embrace lectures on Microscopical Anatomy, Poisons, the Comparative Anatomy of the Nervous System, the Medical Jurisprudence of Midwifery, some of the Diseases of Females, and Clinical Instruction at the Marine Hospital. No charge is made for admission to these lectures, and as they embrace some subjects not fully discussed in the regular course, it is believed students will find it profitable to attend them.

